# Auricular acupuncture for shoulder pain: A protocol for systematic review and meta-analysis

**DOI:** 10.1097/MD.0000000000025666

**Published:** 2021-04-30

**Authors:** Xinju Hou, Wei Xiong, Xingzhen Lin, Yu Zhu, Ruifeng Yang, Jiayang Huang, Ziyin Chen, Hongmei Ma

**Affiliations:** a Nanchang Hongdu Hospital of TCM; b Guangzhou University of Chinese Medicine, Guangzhou, China.

**Keywords:** auricular acupuncture, meta-analysis, shoulder pain, systematic review

## Abstract

**Background:**

Shoulder pain is a common problem in outpatient medical practice. Recent studies show that acupuncture has therapeutic effect on releasing symptoms of shoulder pain. The aim of this systematic review and meta-analysis is to access the efficacy and safety of auricular acupuncture for shoulder pain.

**Methods:**

Eight databases will be searched for randomized controlled trials of auricular acupuncture in the treatment of shoulder pain with retrieval time up to September 2020, including PubMed, Embase, The Cochrane Library, Web of science, CNKI, VIP, CBM, and Wangfang Data databases. We will evaluate the methodological quality of the included studies by using Cochrane Risk of Bias tool and conduct data analysis with Review Manager Software.

**Results:**

The results of this study will be disseminated through a peer-reviewed journal publication.

**Conclusion:**

The systematic review will provide up-to-date evidence for the efficacy and safety of auricular acupuncture in treating shoulder pain.

**PROSPERO registration number:**

CRD 42021238797

## 1 Introduction

The shoulder joint is one of the most movable joints in the human body. It can move in multiple directions, such as up and down, forward and backward, abduction and retraction, and so on.^[1]^ Shoulder pain is a common problem in outpatient medical practice, which is also the third most common musculoskeletal condition the physicians are presenting within primary health care.^[2–4]^ There are many causes of shoulder pain: internal problem such as a sprain or a simple muscle strain can cause a large tear in one of the shoulder stabilizer muscles, external problem can also result in shoulder pain. Some of the shoulder pathologies may result in chronic pain.^[3]^ The most common diagnosis of patients with shoulder pain is subacromial pain syndrome, including rotator cuff syndrome, bursitis, and tendinitis. Besides, there are many more serious shoulder pathologies that may be neurological or injurious, causing severe pain and affecting life.^[5]^

According to reports, the prevalence of shoulder pain in the general population varies from 7% to 30%, which increases with age, and the prevalence of women is higher than that of men. Most of the etiologies of shoulder pain can be fully treated by nonsurgical methods such as oral analgesics, activity and work modification, anti-inflammatory medication, physical therapy, exercise therapy, manual therapy, and subacromial corticosteroid injection. The new guidelines encourage nonpharmacological therapies as the first-line treatment for various of pain.^[6]^ Most people with shoulder pain can improve with physical therapy.^[7]^ However, surgeries are necessary in some conditions to correct and restore the shoulder function. But more and more evidence shows that compared with simplex physiotherapy, surgery cannot bring better patient prognosis.^[1–4]^ Compared with those conservation therapies such as physical therapy, exercise therapy, and manual therapy, surgery is likely to be more risky and expensive. In addition, especially for long-term treatment, corticosteroid injection brings some negative effects on patients.^[8]^

Auricular acupuncture is a kind of microneedle therapy. Because of its simple operation, long-lasting curative effect, nonaddictive, and no toxic side effects, auricular acupuncture is widely accepted by patients as a treatment routine in medical practice. The theory of Traditional Chinese Medicine believes that auricular acupuncture can regulate Yin and Yang viscera through the meridian in order to restore the balance of yin and Yang. However, western medicine considers that nerves are the main connections between the auricle and the internal organs. By stimulating the nerves on the auricle, the auricular acupuncture has a 2-way regulating effect to correct the imbalance of body to achieve the purpose of preventing and curing diseases.^[9,10]^ Auricular acupuncture analgesia is a major feature of auricular acupuncture, which has been confirmed by a large number of clinical practices. Several studies have found that various types of acupuncture can even relieve pain better than analgesics.^[6]^ It is used to control post-traumatic pain, relieve chronic pain of muscles and joints, women's special physiological pain, cancer pain, and other internal organ pain.^[11]^

Although there are many meta-analysis about the utilization of auricular acupuncture, treatment of shoulder pain is rarely covered. This study aims to access the effectiveness and safety of auricular acupuncture for shoulder pain.

## 2 Methods

### 2.1 Study registration

The protocol report has been registered with the registration number CRD 42021238797. The systematic review will be structured stick to the Preferred Reporting Items for Systematic Reviews and Meta-analysis (PRISMA) statement.

### 2.2 Inclusion criteria

#### 2.2.1 Study type

Clinical randomized controlled trials in Chinese or English only, whether take measures to use blinding and allocation concealment or not.

#### 2.2.2 Intervention

The intervention of the experimental group included ear needle, ear bean, electroacupuncture, fire needle, warm needle, acupoint injection, auricular bloodletting, among others. The intervention of the control group was not limited.

#### 2.2.3 Participants

Participants with shoulder pain that is caused by local lesion of the shoulder joint rather than any other primary disease, and the age, sex, and course of disease of the patients are not limited.

### 2.3 Exclusion criteria

1. Duplicate articles.
2. Unable to get data or data is incomplete.
3. Nonrandomized controlled trials, case report, review article, and animal experiment.
4. The main intervention of the experimental group was not auricular acupuncture but body acupuncture or others.

### 2.4 Outcomes

The effective rate judged by the improvement degree of shoulder joint pain before and after treatment.

### 2.5 Search method

We will comprehensively retrieve PubMed, The Cochrane Library, Embase, Web of science, China journal full-text database (CNKI), Chinese biomedical literature database (CBM), China academic journal database (Wanfang Data), and Chinese journal database (VIP) a total of 8 databases, self-built database retrieval time up to September 2020. We will use a combination of medical subject headings terms and free words to search the articles. Subject words include “auricular acupuncture,” “shoulder pain” and free words include “acupunctures, ear,” “ear acupuncture,” “acupuncture, auricular,” “acupunctures, auricular,” “auricular acupunctures,” “ear acupunctures,” “shoulder pain,” “pain, shoulder,” “pains, shoulder,” “frozen shoulder,” “leaky shoulder wind,” “shoulder periarthritis,” “bursitis of shoulder,” “subacromial impingement syndrome,” At the same time, references of included articles will be manually retrieved. We will adjust different search strategies for different databases. Examples of PubMed and China journal full-text database (CNKI) search strategies are presented in Supplement 1, 2.

### 2.6 Study selection and data extraction

#### 2.6.1 Study selection

**Figure 1:**
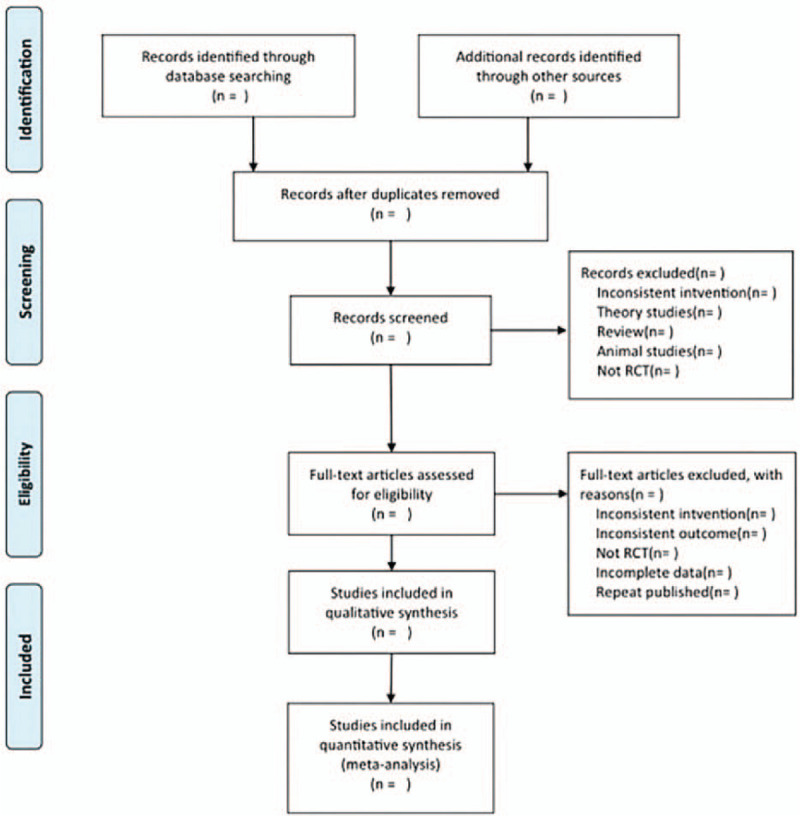
PRISMA flow chart of study selection process.

We will follow PRISMA 2009 Flow Diagram for study selection. We will filter the studies level by level by eliminating duplicates, reading titles and abstracts, and reading the full text. The study screening will be completed independently by 2 researchers, and the third researcher will be responsible for the judgment in case of differences. The literature screening flow chart is shown in Figure 1.

#### 2.6.2 Data extraction

We will use a homemade table to collect the following data included in the article.

1. First author and time of publication, publish journals, and so on.
2. Sample size, sex ratio, age, course of disease, course of treatment, country, etc. of the experimental group, and the control group, among others.
3. Specific interventions for the experimental and control groups, among others.
4. Outcome indicators include primary outcome indicators and secondary outcome indicators.

### 2.7 Risk of bias assessment

The 2 researchers will independently use Cochrane Risk of Bias Tool to assess risk bias in 7 areas: generation of random-allocation methods, allocation concealment, application of blind methods, completeness of outcomes, selective reporting, and other bias risks. The risk bias of included articles will be divided into 3 levels: high, unclear, or low. A third researcher will make the judgment if 2 researchers disagree on an assessment.

### 2.8 Evidence assessed

We will use the internationally accepted Grades of Recommendations Assessment, Development and Evaluation (GRADE) to assess the quality of evidence for the article, which is divided into 4 levels: high, medium, low, and very low.

### 2.9 Data analysis

The article will be conduct with Revman5.3.0 recommended by Cochrane Collaboration. *χ*^2^ test and *I*^*2*^ will be used to analyze the heterogeneity differences among the included articles. When *P* < .05, *I*^*2*^ > 50% the fixed-effect model is used; otherwise, the random-effect model is used. Dichotomous variables will be expressed as relative risk and their 95% confidence interval (95% CI), whereas continuous variables were expressed as mean difference or standardized mean difference and their 95%CI. We will conduct subgroup analysis or sensitivity analysis for studies with large heterogeneity to reduce the impact of heterogeneity differences.

### 2.10 Ethics

As the data source of this study is from the literature of major databases, it does not involve the recruitment of patients or the collection of personal information, so the sanction of the ethics committee is unnecessary.

## 3 Discussion

Shoulder pain is a common complaint in the general population. Many more serious shoulder pathologies, such as hemiplegic shoulder pain, not only adversely affect the physical function but also lower quality of life^[12]^. Although there are some employed forms of treatment for shoulder pain, disease-modifying treatment strategies that have long-lasting curative effect and no toxic side effects for shoulder pain are required.

Auricular acupuncture as an effective technique of TCM has been widely accepted by patients in medical practice^[13]^. According to several studies, auricular acupuncture is able to relieve various kinds of pain, such as cancer pain, chronic back pain, acute pain, and so on.^[14–16]^ Compared with commonly employed forms of treatment for shoulder disorders, auricular acupuncture has the advantage of simple operation, long-lasting curative effect, nonaddictive and no toxic side effects. In this regard, auricular acupuncture may have the potential to overcome the limitations of conventional treatment.

The result of this systematic review will provide up-to-date evidence for the efficacy and safety of auricular acupuncture in treating shoulder pain, which may help to establish better methods for clinicians and patients, as well as a reliable reference for further study.

However, there might be some potential limitations in this systematic review: low quality of original researches, different dosage and frequency of intervention, various duration of disease, language restriction, and so on.

## Author contributions

**Conceptualization:** Xinju Hou, Xingzhen Lin.

**Data curation:** Yu Zhu, Ruifeng Yang, Jiayang Huang, Ziyin Chen.

**Formal analysis:** Yu Zhu, Ruifeng Yang, Jiayang Huang.

**Investigation:** Hongmei Ma.

**Resources:** Hongmei Ma.

**Software:** Wei Xiong.

**Supervision:** Xinju Hou, Hongmei Ma.

**Writing – original draft:** Xinju Hou, Wei Xiong, Xingzhen Lin.

**Writing – review & editing:** Xinju Hou, Wei Xiong, Xingzhen Lin.

## Supplementary Material

Supplemental Digital Content

## Supplementary Material

Supplemental Digital Content
