# Supplementary material for: Auricular acupuncture for shoulder pain: A protocol for systematic review and meta-analysis
Source: Medicine (Baltimore). 2021 Apr 30;100(17):e25666. doi: 10.1097/MD.0000000000025666 (PMC8084003; doi:10.1097/MD.0000000000025666)
Supplement: Supplemental Digital Content [file medi-100-e25666-s001.docx]

**Supplement 1. Search strategy used in PubMed database**

**Number Search terms**

#1 auricular acupuncture [Mesh]

#2 auricular acupuncture OR acupunctures, ear OR ear acupuncture OR acupuncture, auricular OR acupunctures, auricular OR auricular acupunctures OR ear acupunctures [Title/Abstract]

#3 shoulder pain [Mesh]

#4 shoulder pain OR pain, shoulder OR pains, shoulder OR frozen shoulder OR leaky shoulder wind OR shoulder periarthritis OR bursitis of shoulder OR subacromial impingement syndrome [Title/Abstract]

#5 randomized controlled trial OR controlled clinical trial OR randomized [Title/Abstract]

#6 #2 And #4 And #5
