# Supplementary material for: Auricular acupuncture for shoulder pain: A protocol for systematic review and meta-analysis
Source: Medicine (Baltimore). 2021 Apr 30;100(17):e25666. doi: 10.1097/MD.0000000000025666 (PMC8084003; doi:10.1097/MD.0000000000025666)
Supplement: Supplemental Digital Content [file medi-100-e25666-s002.docx]

**Supplement 2. Search strategy used in CNKI database**

SU=耳针+耳穴+耳压+耳穴贴压+耳穴埋豆+耳穴按压+耳穴压籽+耳穴放血+耳穴电针+耳穴注射 AND SU=肩关节疼痛+肩部疼痛+五十肩+冻结肩+肩凝症+漏肩风+肩峰下撞击综合征
